# Pleomorphic Rhabdomyosarcoma Arising from True Vocal Fold of Larynx: Report of a Rare Case and Literature Review

**DOI:** 10.1155/2016/8135967

**Published:** 2016-09-06

**Authors:** Sevdegül Mungan, Selçuk Arslan, Eda Küçüktülü, Şafak Ersöz, Bengü Çobanoğlu

**Affiliations:** ^1^Department of Pathology, Karadeniz Technical University School of Medicine, Trabzon, Turkey; ^2^Department of Otorhinolaryngology, Head and Neck Surgery, Karadeniz Technical University School of Medicine, Trabzon, Turkey; ^3^Department of Radiation Oncology, Kanuni Training and Research Hospital, Trabzon, Turkey; ^4^Department of Otorhinolaryngology, Head and Neck Surgery, Kanuni Training and Research Hospital, Trabzon, Turkey

## Abstract

We present an extremely rare case of a pleomorphic rhabdomyosarcoma of the true vocal fold. The histopathological diagnosis was confirmed by immunohistochemistry. The patient was treated with radical surgery including total laryngectomy and radical neck dissection followed by postoperative radiotherapy. The clinicopathologic features of this rare malignancy are discussed together with a review of the literature. This case report and literature review highlights the more favorable prognosis of pleomorphic rhabdomyosarcoma in the larynx than in other locations.

## 1. Introduction

Neoplastic diseases of the larynx of mesenchymal origin are rare. Over 95% of the malignant neoplasms of the larynx are of squamous histology. Primary laryngeal sarcomas are rare, representing less than 1% of all malignant tumors of the larynx. More than 50% of laryngeal sarcomas are fibrosarcomas, followed by chondrosarcomas, osteosarcomas, leiomyosarcomas, liposarcomas, and rhabdomyosarcomas [1]. While rhabdomyosarcomas (RMSs) account for 40% of all head and neck sarcomas, primary pleomorphic RMS of the larynx seems to be exceedingly rare with only a few case reports recently published [1]. The following three histological subtypes have been identified: embryonal botryoid types, alveolar histotypes that generally affect young children and adolescents, and pleomorphic subtypes that usually affect adult patients. Rhabdomyosarcoma of the larynx was first described by Glick in 1944 [2]. Less than 3% of the cases of cervicocephalic RMSs have been diagnosed in the larynx [3]. Laryngeal RMSs follow a less aggressive course compared to other cervicocephalic rhabdomyosarcomas, and this characteristic feature provides an advantage in locoregional control, disease-free survival, and overall survival [4]. According to Intergroup Rhabdomyosarcoma Studies (IRS) III and IV, the average 5-year survival was reported to be 90% in patients with no residual disease after surgery, 80% in patients with microscopic residual disease, and 70% in patients with macroscopic disease [5]. We report a rare case of laryngeal pleomorphic rhabdomyosarcoma in a 64-year-old patient together with a pertinent review of the literature.

## 2. Case Report

**Figure 1:**
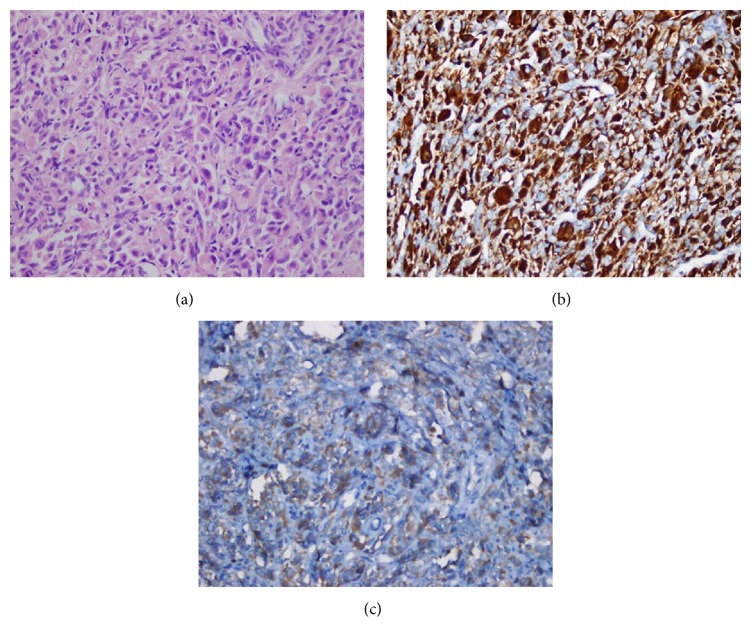
(a) Photomicrograph demonstrating significant pleomorphism and variable dimension of rhabdomyoblastic cells with abundant eosinophilic cytoplasm and eccentric nuclei (HE ×400). (b) The neoplastic cells showing strong desmin positivity by immunohistochemical reaction (desmin ×400). (c) The neoplastic cells with nuclear staining for myogenin (myogenin ×400).

**Figure 2:**
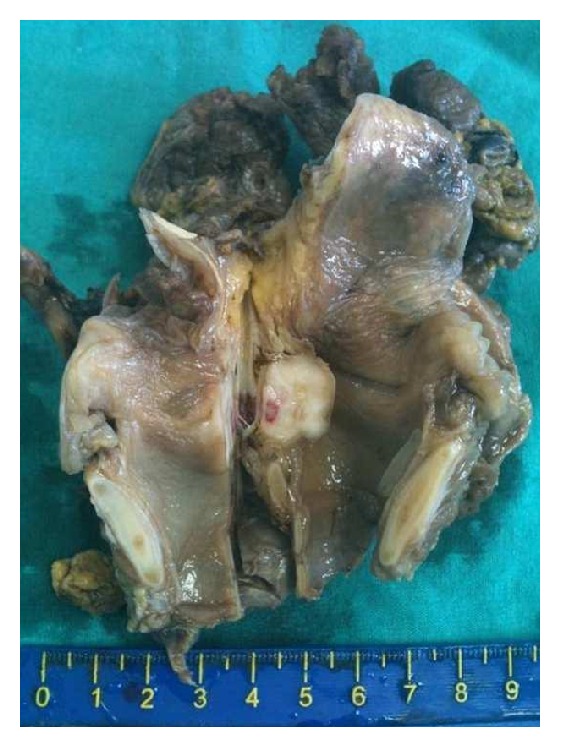
Macroscopic section of the total laryngectomy specimen showing the solid tumoral lesion in the left vocal cord.

A 64-year-old man was referred to the otorhinolaryngology outpatient clinic of the Karadeniz Technical University Medical School with a 2-month history of hoarseness. He had not been smoking for 5 years but had smoked for 10 years previously. He denied excessive alcohol consumption. A laryngeal examination revealed thickening in the left true vocal fold. There was fixation of the left vocal cord and approximately a 3 cm tumor arising from the left true vocal fold invaded the left arytenoid and occupied the ventricle. A biopsy was taken from the lesion with suspension microlaryngoscopy under general anesthesia. A histopathological diagnosis of pleomorphic rhabdomyosarcoma was made based on the detection of fused cells of variable dimensions with eosinophilic cytoplasm, which were irregular and hyperchromic (see Figure 1(a)). They also had large or multiple nuclei. The neoplastic cells expressed desmin (see Figure 1(b)), actin, and myogenin (see Figure 1(c)) in immunohistochemical testing, confirming the diagnosis of rhabdomyosarcoma. The immunostainings with p63, p40, EMA, and cytokeratin-7 were all negative excluding the diagnosis of sarcomatoid carcinoma. Computerized tomography (CT) examinations of the abdomen and thorax revealed no metastases, and no lymphadenopathy was detected in the neck. The tumor was clinically staged as T3N0M0 (stage III) glottic cancer. The patient underwent total laryngectomy (see Figure 2) and left radical neck dissection with an uneventful postoperative recovery. A histological examination of the total laryngectomy specimen confirmed the diagnosis of pleomorphic rhabdomyosarcoma and excised lymph nodes were reported to have reactive hyperplasia. Because the surgical margins were in close proximity to the neoplastic cells (<1 cm), 3-dimensional conformal radiotherapy was performed with 6 MV photons in an Elekta Synergy Platform linear accelerator. Grade III-IV mucositis did not develop, as the patient received Ethyol and glutamine during the therapy. The patient decided not to receive chemotherapy after the evaluation of the tumor size, tumor grade, and lymph node involvement. The patient has continued monthly follow-up visits and has remained disease-free for 2 years after the histological diagnosis.

## 3. Discussion

There are three major categories of RMS, which include pleomorphic, embryonal, and alveolar RMS. The botryoid type is also mentioned in the literature, but it is accepted as a variant of the embryonal category. Pleomorphic RMS is considered to be the least common of the three categories of tumor.

A literature review revealed 21 cases of laryngeal RMS to date. In the last 15 years, only 6 cases of pleomorphic RMS of the larynx have been reported. Prgomet et al. [6] illustrated a case of pleomorphic RMS treated with CO_2_ laser cordectomy and chemotherapy; Shayah et al. [7] reported a case in a 68-year-old male; Schrock et al. [1] described 2 cases of adult pleomorphic RMSs and Pittore et al. [8] reported a 75-year-old male with laryngeal pleomorphic RMS. Chiramel et al. [9] recently described a case of pleomorphic RMS of the supraglottic larynx in a 70-year-old man. According to the literature, pleomorphic RMSs are almost exclusively found in adults, while alveolar and embryonal types are more commonly encountered in children and adolescents [10]. The reported cases in the literature comprised adults ranging from 33 to 77 years of age, with only one exceptional pediatric case reported by Dodd-O et al. [4]. The patient was 5 years old and was cured with total laryngectomy with no evidence of disease after 18 years of follow-up (Table 1). Laryngeal pleomorphic RMSs are found more often in males than in females. There have been 3 cases of female patients [7, 11, 12] reported in the literature versus 19 cases of male patients, including our case.

Differential diagnosis of head and neck RMSs includes lymphoma, neuroblastoma, retinoblastoma, hemangioendothelioma, melanoma, fibrosarcoma, granular cell myoblastoma, and rhabdomyoma. In the current case, sarcomatoid carcinoma (spindle cell carcinoma) should also be considered in the differential diagnosis. Differentiating RMS from liposarcoma or malignant fibrous histiocytoma can be challenging; therefore, a diagnosis of pleomorphic RMS should not be made unless there is incontrovertible evidence of skeletal muscle differentiation in the form of cross-striations or if specific immunohistochemical markers are demonstrated [10]. In the current case, the diagnosis was confirmed by immunohistochemistry. The neoplastic cells expressed desmin, a muscle-specific protein found in cardiac, skeletal, and smooth muscles, as well as actin and myogenin, which are highly sensitive and specific markers for RMSs. The immunostainings with p63, p40, EMA, and cytokeratin-7 were found to be negative excluding the diagnosis of sarcomatoid carcinoma.

Several prognostic indicators have been identified, which include age, tumor location, and histotype. Hawkins et al. [16] reported a better prognosis for patients aged <20 years, with a tumor size <5 cm, treated with radical surgical excision in the absence of locoregional disease.

The biological course of laryngeal pleomorphic rhabdomyosarcomas has not been clearly defined because of the scarcity of clinical reports. However, a review of the literature indicates that most of these lesions are less aggressive than other RMSs of the head and neck regions in general. One of the cases reported in the literature died of unrelated heart disease, while ten cases had no evidence of disease in follow-up periods ranging from 1 year to 6 years. On the other hand, follow-up period and disease-free survival are not documented for all the cases [9, 11, 13]. Chiramel et al. [9] recommended adjuvant chemoradiotherapy followed by surgery for a patient with pleomorphic RMS of supraglottic larynx involving the posterior pharyngeal wall. Unfortunately, they could not report data regarding the treatment outcome for the patient was lost to follow-up after the initial dose of chemotherapy. Akyol et al. [10] reported a case of laryngeal pleomorphic RMS with a very aggressive course in contrast to other reported cases in the literature. The patient died of disease 8 months later despite radical surgery involving total laryngectomy and neck dissection followed by radiotherapy. In our case, the patient has remained disease-free for 24 months after the surgery.

RMSs are locally aggressive and have a tendency to metastasize by both lymphatic and hematological routes, mostly to lung, liver, and bone [8]. Therefore, during follow-up, it is essential to plan for staging and imaging, including CT scans and standard thoracic radiographs [8]. Nonetheless, RMSs of the larynx are believed to be less aggressive than RMSs that primarily localize to other cervicocephalic regions [4, 14]. The treatment principle for malignant mesenchymal tumors of the head and neck region includes radical surgery with postoperative RT and/or chemotherapy and laryngeal RMS is also managed in the same way. To control locoregional disease, the surgery must be radical, allowing for the resection of the tumor with wide margins. The choice for surgery depends on various disease factors (site, extent of lesion). Conversely, factors such as age, general health status of the patient, comorbidities, and patient choices can change the treatment approach to more conservative surgeries or nonsurgical therapies such as radiotherapy and/or chemotherapy. In the present case, we decided to perform a total laryngectomy and radical neck dissection considering the mesenchymal origin and advanced stage of the tumor.

Fifteen of the 21 cases in the literature diagnosed as pleomorphic RMS underwent cold-knife laryngectomy (total/subtotal, with/without neck dissections). Jahnke and Vogl [15] performed total pharyngolaryngectomies as well as cervical, esophageal, and tracheal resections for the surgical treatment. As for the 2 cases in the literature treated without surgery, one case was treated only with radiotherapy, while the other was treated with radiotherapy and chemotherapy. Schrock et al. [1] treated one case of laryngeal RMS with CO_2_ laser cordectomy with radiotherapy, and another case was treated by Prgomet et al. [6] with CO_2_ laser cordectomy with chemotherapy. The treatment choices of all cases in the literature are listed in Table 1. The current case was treated with surgery (total laryngectomy and left neck dissection) and postoperative radiotherapy. When feasible, the literature supports the additional use of chemotherapy to lessen the occurrence of the systemic spread of the disease. In our case, chemotherapy was not included in the treatment protocol as there was no evidence of extracapsular invasion and metastatic lymph nodes in the surgical specimen.

## 4. Conclusion

Laryngeal pleomorphic RMSs are less aggressive than RMSs arising from other sites of the head and neck and respond well to surgery. Other treatment options such as chemotherapy or radiotherapy are also available. As the number of reported laryngeal pleomorphic RMS cases is limited, this tumor's behavior has not yet been fully elucidated. Therefore, further studies are needed to improve our understanding of the biological behavior of this rare malignancy and to determine the most appropriate therapeutic approach to its treatment.

## Figures and Tables

**Table 1:** Case reports of laryngeal pleomorphic rhabdomyosarcoma.

Number	Year	Author	Age (yrs)/sex	Treatment	FU	Status
1	1964	Filipo and Crifo^*∗*^	53/M	Total laryngectomy, RT	8 m	NED
2	1970	Rodriguez and Ziskind [13]	57/M	Total laryngectomy	?	?
3	1971	Laccourreye et al.^*∗*^	65/M	Total laryngectomy	41 d	DOD
4	1976	Frugoni and Ferlito [14]	33/M	Subtotal laryngectomy, RT	6 y	NED
5	1977	Marasso et al.^*∗*^	65/M	RT + CHT	?	?
6	1977	Lamendola and Buonocore^*∗∗*^	61/M	Total laryngectomy, bilat. neck dissection	1 y	NED
7	1978	Winther and Lorentzen [11]	72/F	Subtotal laryngectomy	?	?
8	1979	Seniukov et al.^*∗*^	55/M	Total laryngectomy, RT	4 m	NED
9	1979	Franz^*∗∗*^	57/M	RT	2 y	NED
10	1987	Dodd-O et al. [4]	5/M	Total laryngectomy	18 y	NED
11	1988	De Agostino et al.^*∗∗*^	70/M	Total laryngectomy	?	?
12	1994	Jahnke and Vogl [15]	45/M	Total pharyngolaryngectomy, cervical oesophageal + tracheal resection, CHT + RT	?	?
13	1996	Da Mosto et al. [3]	69/M	Total laryngectomy, RT	2 y	NED
14	1998	Akyol et al. [10]	68/M	Total laryngectomy, neck dissection, RT	8 m	DOD
15	1998	Ruske et al. [12]	66/F	Total laryngectomy, RT	30 m	NED
16	2006	Prgomet et al. [6]		CO_2_ laser cordectomy, CHT	6 y	NED
17	2007	Shayah et al. [7]	77/F	Total laryngectomy	1 y	NED
18	2007	Schrock et al. [1]	60/M	Total laryngectomy, neck dissection, RT, CHT	20 m	NED
19	2007	Schrock et al. [1]	64/M	CO_2_ laser cordectomy, RT	20 m	NED
20	2010	Pittore et al. [8]	75/M	Partial laryngectomy	9 m	NED
21	2015	Chiramel et al. [9]	70/M	?	?	?
22	Our case	Mungan et al.	64/M	Total laryngectomy, neck dissection, RT	24 m	NED

FU: follow-up; NED: no evidence of disease; DOD: died of disease.

^*∗*^ *Cited by* Dodd-O et al. [4]; ^*∗∗*^ *cited by*Da Mosto et al. [3].
